# Long-Term Dietary Consumption of Grapes Alters Phenotypic Expression in Skeletal Muscle of Aged Male and Female Mice

**DOI:** 10.3390/foods14040695

**Published:** 2025-02-18

**Authors:** Asim Dave, Eun-Jung Park, Sumi Piya, John M. Pezzuto

**Affiliations:** 1 Center for Computational and Integrative Biology, Rutgers University, Camden, NJ 08102, USA; asim.dave@rutgers.edu; 2 College of Pharmacy and Health Sciences, Western New England University, Springfield, MA 01119, USA; eunjung.park@wne.edu; 3 Department of Pathology, UMass Chan Medical School-Baystate, Springfield, MA 01199, USA; sumi.piya@baystatehealth.org; 4 Department of Medicine, UMass Chan Medical School-Baystate, Springfield, MA 01199, USA

**Keywords:** genetic metamorphosis, male/female muscle convergence, KEGG, GO, Reactome analytics

## Abstract

(1) Background: Nutrigenomics investigates how diet influences gene expression and how genetic variation impacts dietary responses. Grapes, rich in phytochemicals, exhibit potential disease-preventive properties through nutrigenomic mechanisms rather than direct chemical interactions. This study aimed to explore the modulation of gene expression in muscle tissue resulting from long-term grape consumption. (2) Methods: A mouse model was employed to assess gene expression in the skeletal muscles of males and females fed a grape-enriched diet versus a bland diet over 2.5 years. Heatmaps and principal component analyses were performed to identify patterns, and pathway analyses using KEGG, GO, and Reactome were conducted. (3) Results: Significant sex-specific gene expression changes were observed, with female phenotypes showing greater alterations and converging toward male-like characteristics. Twenty-five differentially expressed genes associated with muscle health were identified. Up-regulated genes such as *Ahsg*, *Alb*, *Apoa1*, and *Arg1*, and down-regulated genes including *Camp*, *Lcn2*, and *Irf4*, suggest improved muscle function. (4) Conclusions: Long-term grape consumption appears to enhance female muscle traits toward a male-like phenotype, potentially indicating broader health benefits. Further studies and clinical trials are needed to confirm human applicability and the physiological implications of these findings. Nonetheless, this research underscores the role of nutrigenomics in understanding dietary influences on gene expression and sex-specific responses.

## 1. Introduction

With over 600 skeletal muscles distributed throughout the human body, nearly all voluntary or involuntary movements rely on proper function. Examples such as vision, hearing, moving, digestive, and cardiac function are obvious. Conversely, muscle abnormalities, such as fibromyalgia, myopathies, amyotrophic lateral sclerosis (ALS), and cardiovascular disease accentuate the critical importance of proper muscle function. Throughout the world, 10–16% of elderly people are estimated to experience sarcopenia, a progressive loss of muscle mass and function [1]. Further, as a consequence of chronic illness (e.g., kidney disease, heart failure, and rheumatoid arthritis), patients face a high risk of cachexia, i.e., weight loss and wasting, particularly of muscle mass [2]. The comorbidity of cachexia is especially high in cancer patients. About 80% of all cancer patients experience cachexia, and this accounts for more than 25% of all cancer deaths [3].

Over the past few decades, the broad-based influence of diet on health and well-being has been emphasized. While we do not expect diet will greatly affect end-stage or highly advanced disease, large-scale epidemiological studies have clearly indicated that high consumption of fruits and vegetables lowers the risk of dying from cancer, cardiovascular disease, and respiratory disease [4]. Risk reduction plateaued with the daily consumption of about five servings of fruit and vegetables, which is the general dietary recommendation provided by organizations such as the American Health Association (AHA) and the National Cancer Institute (NCI). Rather than, or in addition to, the nutritive value of fruits and vegetables, these types of health benefits are generally associated with ingesting the respective phytochemical constituents [5,6].

Many phytochemicals that are naturally occurring in fruits and vegetables have been individually studied for the prevention or treatment of human disease. Resveratrol, a component of grapes and wine, is a key example. First reported by our group as a potential cancer chemopreventive agent functioning by pleotropic mechanisms of action [7], resveratrol has subsequently been investigated in hundreds of model systems and clinical trials [8]. Notably, however, the concentrations and doses of resveratrol far exceed those that can realistically be achieved by dietary consumption. Therefore, the material must be viewed as a natural product drug or dietary supplement, which is a pattern mirrored by most other phytochemicals associated with fruits and vegetables.

Accordingly, it may be suggested that studies performed with high doses of individual phytochemical constituents have intrinsic value but are not a good representation of responses that might be anticipated by normal dietary habits. Rather, any relevant responses mediated by a whole food would be expected to result from consumption of the milieu of endogenous constituents, likely numbered in the thousands, as well as a myriad of other factors, such as plant growth conditions, storage, processing, digestive, and absorption properties, etc.

Taking these issues into account, we have focused on the biologic potential of the grape as a whole food. In addition to resveratrol, the grape is known to contain over 1600 phytochemicals [9], the sum total of which would certainly be expected to mediate a unique response relative to any single component. To assure scientific rigor and the reproducibility of results, a standardized surrogate representative of the whole grape has been employed in numerous studies designed to assess the possible influence of grapes on health. Interestingly, effects have been observed that are relevant to a number of human conditions involving the heart [10], gastrointestinal system [11], bladder [12], cognition [13], vision [14], skin [15], etc. Various mechanisms may apply, including modulation of the human microbiome [16,17].

Rather than concentrating solely on individual mechanisms, which are typically reported using a high concentration of individual agents (such as antioxidant activity, modulation of specific pathways like NF-ĸB, enzyme inhibition, etc.), we have been exploring the potential of whole grapes to modulate phenotypic expression. Our assumption is that results obtained using this nutrigenomic approach more fully represent the physiological response to diet, and the factors that are modulated function in a ‘catalytic’ manner. As such, using dietary dose regimens of grapes that realistically correlate with human consumption levels, we have demonstrated alteration of genetic expression in various mouse organs [18], as well as an influence on kidney health [19]. Further, when given concomitantly with a high-fat diet, grape consumption improves cognition [20], retards fatty liver development [21], and enhances lifespan [21]. Notably, changes in gene expression correlate with alteration of the metabolome [22].

Currently, we focus our attention on the potential of grape consumption to affect muscle. As noted above, loss of muscle mass and function is commonly associated with the aging process, and sarcopenia is expected to increase in the future, along with the increasing population of elderly people. Preventative strategies primarily rely on exercise and high-protein diets [23]. In addition, some individual phytochemicals associated with the grape, including resveratrol [24,25], quercetin [26], and catechins [27], have shown beneficial effects on skeletal muscle health. We were curious if long-term grape consumption would modulate gene expression in the skeletal muscle of male and female mice maintained over a period of 2.5 years, which correlates with an age of approximately 80 years in a human [28].

In addition, a unique aspect of the current work is the investigation of nutrigenomic diversity in the context of sex. As well described by Corella et al. [29], the potentially profound influence of sex on nutrigenomic response is extensive but often goes unreported. When sex is considered, such as with consumption of the Mediterranean diet by male and female subjects [30], gene expression responses vary between the two groups. The possibility of such variation resulting from hormonal and genotype (XX vs. XY) differences is intuitive, but the situation is even much more complex. For example, as reported by Taglia et al. [31], sex-heterogeneous single-nucleotide polymorphisms (sex-het SNPs) disproportionately influence gene expression, including at sites in/near genes with roles in skeletal and muscle development.

As reported herein, sex has a strong influence on gene expression in skeletal muscle provoked by the addition of grapes to an otherwise bland isocaloric diet. Compared with the standard diet, following grape consumption, phenotypic expression in the male and female groups becomes much more homogeneous.

## 2. Materials and Methods

### 2.1. Animals and Diets

The animal models, diets, and experimental design were described previously [19]. This study was approved by the Institutional Animal Care and Use Committee (IACUC) of Baystate Health, Springfield, MA (protocol number 1736198-2, approved 20 April 2021).

As detailed in Table 1, custom isocaloric diets were formulated and produced by Envigo (Madison, WI, USA): 4% fat standard diet (SD, TD.160157) and a 4% standard diet supplemented with 5% grape powder diet (SDG, TD.160158). Freeze-dried grape powder is used as a surrogate for whole grapes. The powder was prepared, analyzed, and verified to be contamination-free under the auspices of the California Table Grape Commission (Fresno, CA, USA) [32]. Provision of this standardized material helps to ensure long-term consistency across experimental and clinical studies.

In clinical trials with humans, the daily consumption of two servings of grapes, which is around one and a half cups of fresh grapes or 252 g, equates to 46 g of the freeze-dried whole table grape powder from the California Table Grape Commission [32,33]. Based on relative body weight, daily food consumption, and standard mammalian conversion factors [34], the addition of 5% grape powder to a mouse diet roughly equates to the human consumption of two servings of grapes per day. The addition of grape powder to the isocaloric diet does not significantly affect food consumption rates.

### 2.2. Experimental Design

**Figure 1:**
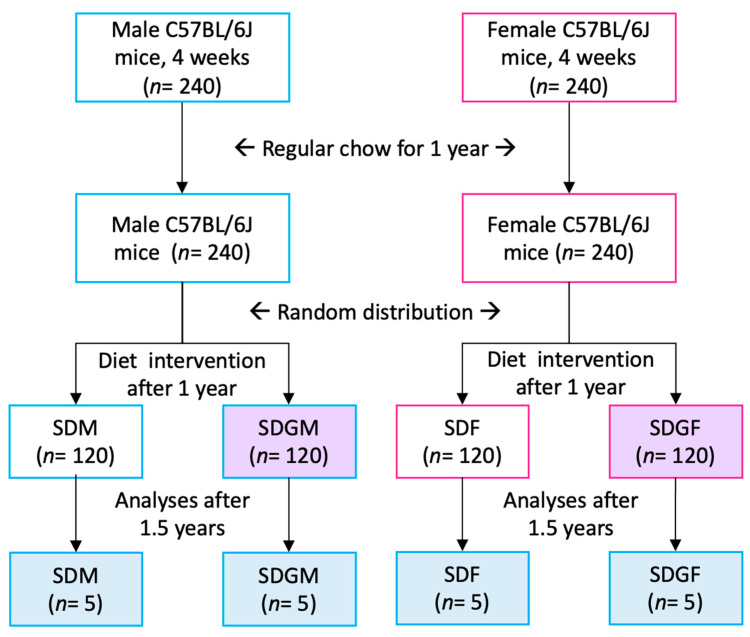
Overall experimental protocol yielding the laboratory animals used in the current investigation.

The overall experimental design is illustrated in Figure 1. This work was a subset of a parent study in which a total of 480 C57BL/6J mice (240 males and 240 females), obtained from The Jackson Laboratory (Bar Harbor, ME, USA), were introduced to the standard diet at 4 weeks of age [21]. The sample size of 240 male and 240 female C57BL/6J mice was determined using a power analysis conducted with G*Power (effect size = 0.3; alpha = 0.05; degrees of freedom = 1; version 3.1.9.7). This was a lifetime survival study. A reserve group of 20 mice per cohort was maintained to replace any that were removed due to abnormal conditions, so that only natural deaths were considered in the survival analysis, as well as having sufficient mice for the current study.

At 1 year, the mice were randomly assigned to four equal groups (120 per group) (Figure 1). Half of the mice continued on the standard diet, while the other half were switched to the grape diet. Mice were housed in HEPA-filtered cages (four per cage), with controlled temperature (21 ± 2 °C), humidity (30–70%), and a 12 h light–dark cycle. The mice had free access to food and water, and each mouse was implanted with an RFID microchip for identification. Body weight was recorded biweekly.

For performing the analyses described herein, five mice were randomly selected from the SDM, SDGM, SDF, and SDGF groups at 2.5 years of age. The duration of 2.5 years was chosen to mirror old age, since this period in the lifespan of a mouse approximates a human equivalent age of 80 years [28]. Objectives of the work were to assess how control animals given what may be termed a “bland” diet might differ from animals given the same diet supplemented with grapes, and to assess any differences due to sex.

Standard scientific dogma requires a minimal sample size of three to allow the identification of outliers, to perform proper statistical analyses, etc. This may be insufficient in the case of high variability, low statistical power, or increased risk of Type 1 and Type 2 errors. We selected a sample size of five, with the hope of reducing variability and improving power, as well as ethical considerations. In addition, however, the analyses reported herein are resource intensive. Accordingly, this pragmatic aspect needed to be considered. In any case, the sample size proved sufficient, based on the statistical protocols to which the data were subjected.

### 2.3. Tissue Collection

At 2.5 years of age, five mice from each of the four groups were randomly selected for euthanasia following an overnight fasting period. At this time, the age of a mouse roughly correlates with that of an 80-year-old human [28]. Body weights were measured before euthanasia, which was conducted using CO_2_ inhalation followed by cervical dislocation. Thigh muscles were harvested, halved, and promptly preserved in RNAlater™ stabilization solution (ThermoFisher Scientific, Waltham, MA, USA) or fixed in 10% neutral buffered formalin (NBF). Samples in RNAlater^TM^ were stored at −20 °C until RNA extraction.

### 2.4. Histopathological Examination of Muscles

After fixation in 10% NBF, muscle samples were transferred to 70% ethanol, dehydrated through graded ethanol, cleared in xylene, and paraffin-embedded using a Leica ASP300S (Leica Biosystems, Nussloch, Germany). Sections (4 µm) were cut, deparaffinized, rehydrated, and stained with hematoxylin and eosin (H&E) or Masson’s trichrome. After dehydration and clearing, slides were mounted with Cytoseal 60.

### 2.5. RNA Extraction and Sequencing

Tissue homogenization was performed using 750 μL of QIAzol (Qiagen, Germantown, PA, USA) following the manufacturer’s protocol in conjunction with the RNeasy 96 Universal Tissue Kit (Qiagen, Germantown, PA, USA). The quantity and quality of the extracted RNA were assessed using a BioSpec-nano spectrophotometer (Shimadzu, Tokyo, Japan), while RNA integrity was evaluated via QIAxcel^®^ capillary electrophoresis (Qiagen). For transcriptome library preparation, RNA samples were quantified, and only those with an RNA integrity number greater than 6 were selected for downstream processing. Poly(A)-mRNA was purified using magnetic beads, followed by cDNA synthesis according to the standard script-sequencing protocol. Library quality was assessed using a Qubit fluorometer (ThermoFisher Scientific) and RT-PCR for quantification before sequencing. The transcriptome yield directly reflected gene expression levels. Sample qualification criteria included DNA quantification using the Qubit 2.0 DNA HS Assay (Life Technologies, Grand Island, NY, USA) and quality assessment via 1% standard agarose gel electrophoresis and/or the TapeStation Genomic DNA Assay (Agilent Technologies, Santa Clara, CA, USA). RNA quantity and quality were evaluated using the Qubit RNA HS Assay (ThermoFisher Scientific, USA) and the Bioanalyzer 2100 Eukaryote Total RNA Nano Assay (Agilent Technologies, CA, USA), respectively. Library concentration and quality were further determined using the Qubit 2.0 DNA HS Assay (ThermoFisher Scientific, USA), QuantStudio^®^ 5 System (Applied Biosystems, San Francisco, CA, USA), and the TapeStation High-Sensitivity D1000 Assay (Agilent Technologies, CA, USA).

RNA extraction and sequencing were conducted by Novogene Corporation Inc. (Sacramento, CA, USA). The library construction utilized paired-end (PE) 150 bp sequencing on the Illumina HiSeq platform, generating approximately 20 million raw reads per sample. Raw sequencing data in fastq format were initially processed using in-house Perl scripts to generate clean reads by removing adapter-containing sequences, poly-N reads, and low-quality reads. During this step, quality metrics such as Q20, Q30, and GC content were calculated to ensure data integrity, and all downstream analyses were based on the resulting high-quality clean data. To minimize the impact of low-quality reads on downstream analysis, filtering was performed by eliminating reads with adapter contamination, reads with more than 10% uncertain nucleotides (N > 10%), and reads in which over 50% of bases had a quality score below 5. Following gene expression quantification, statistical analysis was conducted to identify differentially expressed genes across conditions. This involved normalizing the raw read counts to account for sequencing depth, a process carried out using the DESeq method.

### 2.6. Pathway and GO Term Enrichment Analyses

Pathway analyses were performed using the clusterProfiler [35] package(4.14.4) for enrichment analysis, including GO terms curated in the gene ontology resource (https://geneontology.org, accessed on 4 November 2024), as “biological process”, “molecular function”, and “cellular component” aspects, KEGG (http://www.kegg.jp/, accessed on: 4 November 2024), and Reactome database (http://www.reactome.org, accessed on: 4 November 2024). Significant enrichment was determined by considering an adjusted *p* value (Padj) < 0.05.

### 2.7. Heatmap Generation

Heatmaps were generated using the pheatmap package in R [36]. FPKM values were first transformed to *z*-scores using zFPKM (1.28.0) [37] to standardize gene expression levels. The pheatmap function was employed to produce a heatmap from the filtered *z*-score matrix. Hierarchical clustering was applied to both rows and columns to group genes and samples based on expression similarity. K-means clustering was applied to explore groupings of genes with similar expression patterns across samples. Further, the cutree function [38] in R was applied for the genes differentially expressed. These clusters were visually inspected and defined based on the structure of the dendrograms.

### 2.8. Principal Component Analysis

Principal component analysis (PCA) was performed on the normalized, log2-transformed gene expression data to identify underlying patterns and reduce dimensionality. The analysis was conducted using the prcomp function from the stats package in R.

### 2.9. Differential Expression Analyses

Differential expression analyses among the four diet groups were performed using the DESeq2 package (1.42.1) [39]. DESeq2 provides statistical routines for determining differential expression in digital gene expression data using a model based on the negative binomial distribution. The resulting *p*-values were adjusted using the Benjamini and Hochberg approach for controlling the false discovery rate (FDR) to yield Padj (*q*-values). Genes with *q* < 0.05 found by DESeq2 and |log2(FoldChange)| ≥ 1 were set as the thresholds for significant differential expression and were assigned as differentially expressed. Further, to quantify gene expression levels, we employed Fragments Per Kilobase of transcript per Million mapped reads (FPKM). The DEG list generated includes genes with FPKM values greater than 1.

### 2.10. Statistical Analyses

Statistical significance was considered by calculating Padj to mitigate the effect of false positives in the analyses. Padj were calculated using the Benjamini and Hochberg method to control the FDR and minimize the likelihood of false positives. Differential gene expression analysis was performed using the DESeq2 package in R, which applies the Benjamini–Hochberg method to adjust *p*-values derived from Wald tests. Padj ≤ 0.05 was applied across the analysis to define statistical significance. Other statistical methods applied during the course of this work are recorded in the text.

## 3. Results

### 3.1. Gross Observations

As detailed in a previous report [19], there was no discernable difference in the weight of the male or female mice, irrespective of dietary grape supplementation, nor were there any significant differences in the weight of the muscle. For the SDM and SDGM, the respective muscle/body weight ratios were 0.013 ± 0.003 and 0.014 ± 0.004 (*p* = 0.85), and for the SDF and SDGF groups the respective muscle/body weight ratios were 0.050 ± 0.071 and 0.046 ± 0.067 (*p* = 0.93). The SDM did not differ from the SDF (*p* = 0.28), nor did the SDGM differ from the SDGF (*p* = 0.32) [19]. It has been reported in the literature that for mice, skeletal muscle as a percentage of body weight does not vary for males and females in all stages of development [40].

### 3.2. Histopathological Evaluation of Muscle Tissues

**Figure 2:**
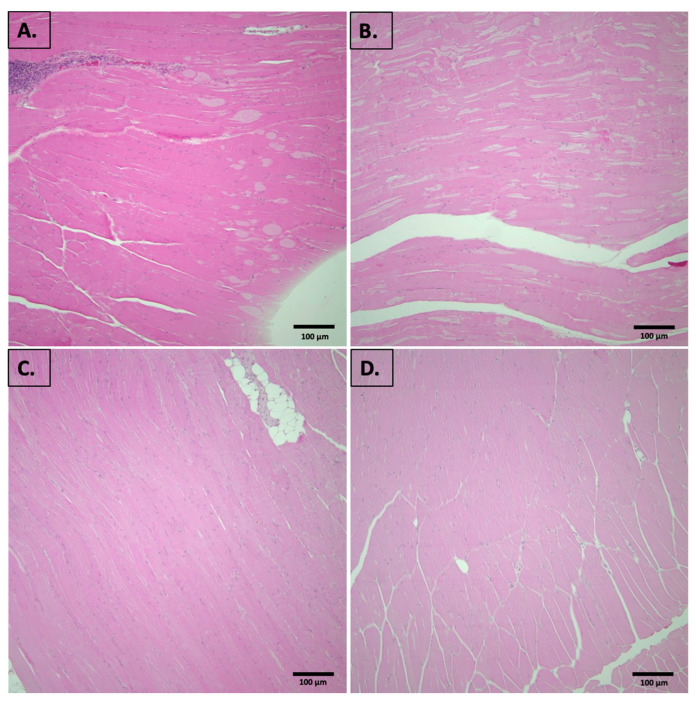
Representative images of mouse muscle from (**A**) male, SDM; (**B**) male, SDGM; (**C**) female, SDF; (**D**) female, SDGF. (**A**) represents chronic perivascular inflammation. (**B**–**D**) represent normal muscle histology with no significant pathological changes. Four-micron sections were stained with H&E. Original magnification, 20X.

Muscle tissues from all designated groups were subjected to histopathological evaluation using both H&E and Masson’s trichrome stains. As illustrated in Figure 2, both male and female mice on the standard diet exhibited predominantly normal muscle histology, with only minimal chronic perivascular inflammation in one male mouse specimen, which was not considered a significant pathological finding. Similarly, male and female mice receiving the grape-supplemented diet displayed normal muscle histology, with no evidence of significant pathological changes. In sum, no meaningful histopathological differences were seen between the groups, either based on sex or diet.

### 3.3. Venn Diagrams

**Figure 3:**
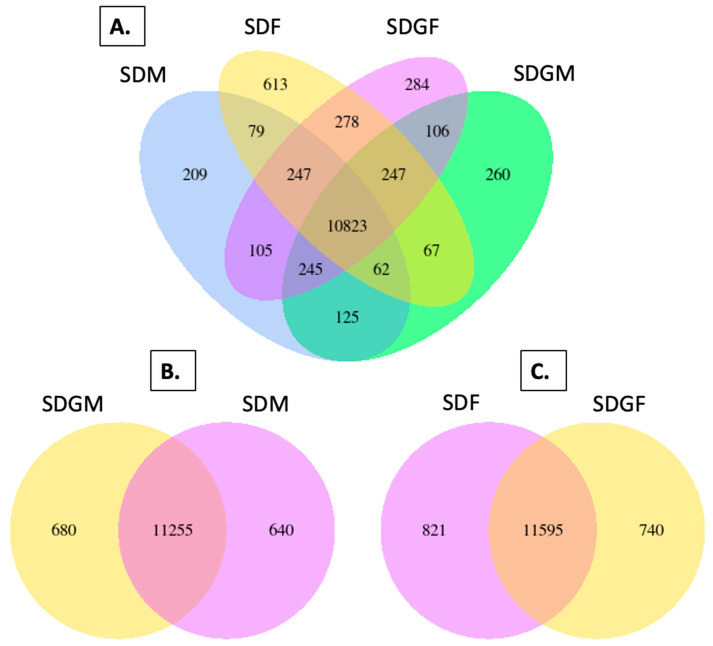
Venn diagrams depicting genes expressed in the muscles of mice provided with standard and grape-supplemented diets. The default threshold of the FPKM value is set to 1 for the selection of the genes for each group. (**A**) Venn diagram of four groups, SDM, SDF, SDGF, and SDGM, with genes co-expressed and uniquely expressed among all groups. (**B**) Venn diagram of genes expressed by SDGM and SDM. (**C**) Venn diagram of genes expressed by SDF and SDGF.

While histopathological analysis did not reveal significant differences between groups, gene expression analysis provided deeper insights into potential biological variations. To quantitatively analyze the distribution of genes expressed by the different groups, Venn diagrams were constructed to visualize shared and unique gene sets. Figure 3A illustrates the gene distribution across all four groups. While the majority of genes (>10,000) were shared among all four groups, unique gene sets were shared between two or three groups, and each individual group demonstrated gene sets unique to the respective group. The abundance of unique genes was relatively consistent across groups, except for SDF, which showed the highest number of unique genes. Figure 3B,C illustrate comparisons between grape and standard diets for males and females, respectively. As expected, the majority of genes were common irrespective of diet. However, both the male and female groups did show significant variation when comparing those provided with the standard diet versus those provided with the grape-supplemented diet.

### 3.4. Volcano Plots

**Figure 4:**
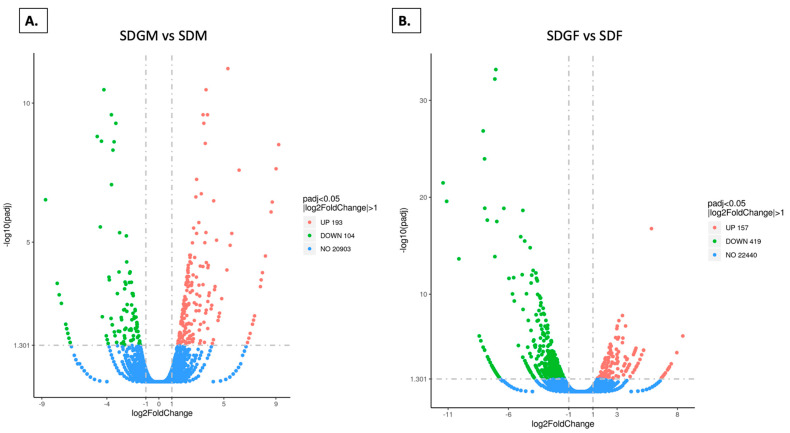
Volcano plots depicting alteration of gene expression in the muscles of male or female mice provided with standard or grape-supplemented diets. (**A**) Volcano plot of SDGM vs. SDM showing up-regulated (red dots), down-regulated (green dots), and unaltered (blue dots) genes. (**B**) Volcano plot of SDGF vs. SDF showing up-regulated (red dots), down-regulated (green dots), and unaltered (blue dots) genes. In each case, the threshold for differentially expressed genes was set as |log2(fold-change)| > 1 and −log10(Padj) > 1.3 (Padj < 0.05) (separated by dotted lines).

To further assess the extent of gene expression differences between dietary groups, volcano plots were generated to highlight significantly enriched genes. To evaluate gene expression differences between the male or female groups provided with standard or grape-supplemented diets, volcano plots were constructed with threshold values to illustrate enriched genes and allow visualization of the number of genes up- and down-regulated. As shown in Figure 4A, a total of 21,200 genes were observed when comparing the SDGM and SDM groups, with 193 genes up-regulated and 104 down-regulated. For the SDGF and SDF group comparisons, as shown in Figure 4B, a total of 23,016 genes were identified, with 157 genes up-regulated and 419 down-regulated.

### 3.5. Comparative Heatmaps

**Figure 5:**
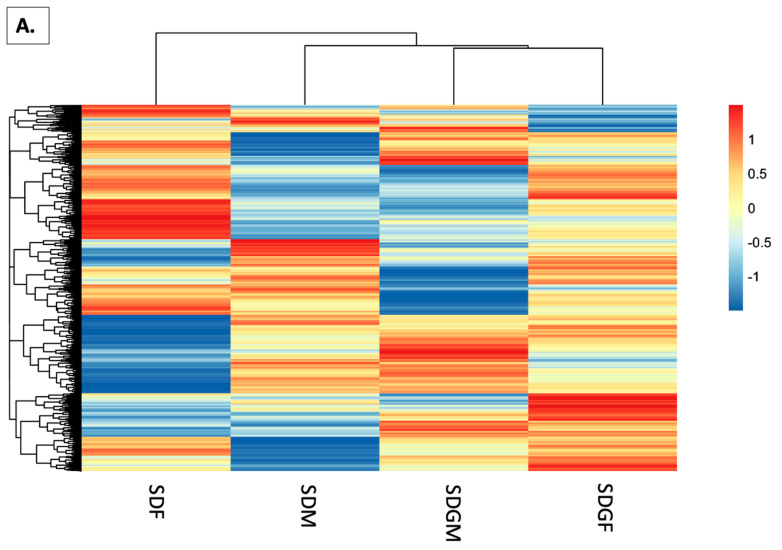

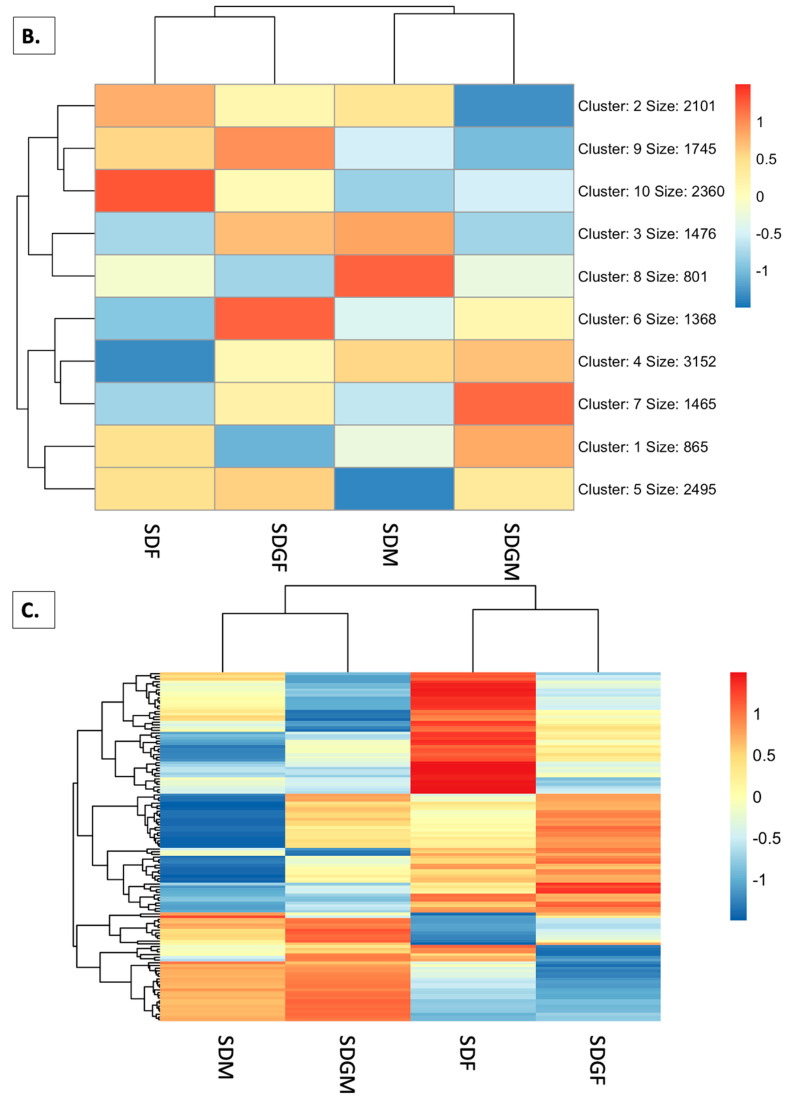

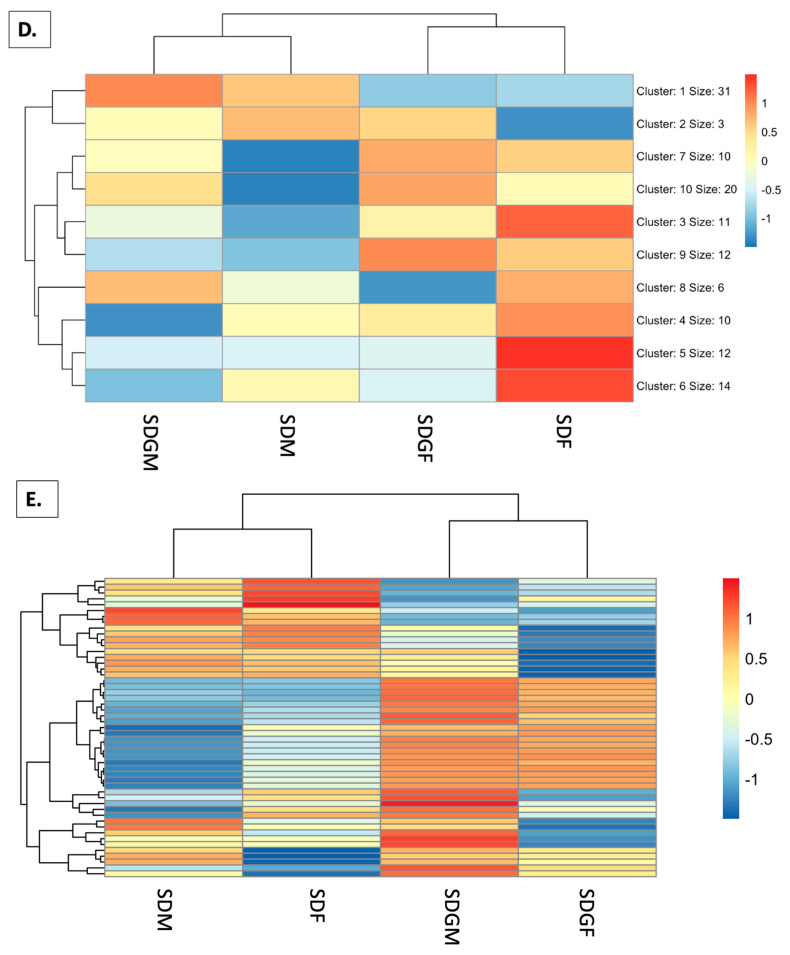

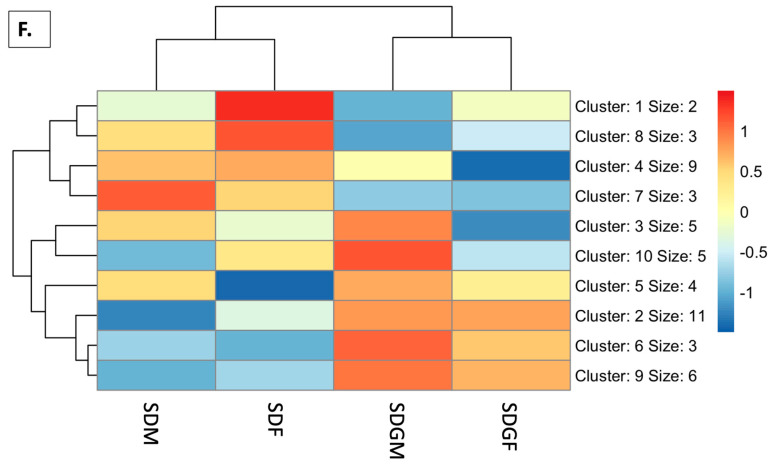
Heatmaps illustrating gene expression in the muscles of male and female mice provided with control and grape-supplemented diets. The changes in expression are represented by the *z*-score. (**A**) Heatmap showing expression changes for global genes in the muscles. Hierarchical clustering shows distinct expression patterns, with male and female groups differing under standard diets. Grape supplementation alters clustering, aligning SDGF more closely with SDGM than SDF. (**B**) Genes from the global expression heatmap (Figure 5A) are organized into 10 clusters using k-means clustering. The scale on the right indicates relative enrichment levels for each cluster. Grape supplementation induces distinct gene expression changes, impacting both sexes similarly. (**C**) Heatmap showing the expression of the DEG list identified from the overlap between SDM vs. SDF and SDGM vs. SDGF comparisons. Hierarchical clustering primarily groups samples by sex, linking SDF with SDGF and SDM with SDGM. (**D**) Expression changes for the DEG list identified from the overlap between SDM vs. SDF and SDGM vs. SDGF comparisons represented by clusters of genes generated using k-means analysis. Relative enrichment levels are represented using an 11-color palette and assigned quantitative values (−1.5 to +1.5). Quantitative analysis reveals net differences of 7.4 units (SDF vs. SDM) and 1.4 units (SDGF vs. SDGM), showing convergence between sexes upon grape supplementation. (**E**) Heatmap showing the expression levels of DEGs identified from the overlap between SDGM vs. SDM and SDGF vs. SDF comparisons. Hierarchical mapping links groups by diet (SDGM and SDGF). (**F**) Expression changes of the DEG list from the overlap between SDGM vs. SDM and SDGF vs. SDF comparisons represented by clusters of genes generated using k-means analysis and quantitatively analyzed using the 11-color palette. There is a net difference of 4.7 units (SDM vs. SDGM) and 2.7 units (SDF vs. SDGF). The sum of these values matches the 7.4-unit difference seen in SDF vs. SDM. The combined movement shows increased similarity between sexes under grape supplementation.

To examine relative gene expression patterns with the four groups, heatmaps were generated to compare group differences, visualize clustering of similar gene sets, and examine hierarchical mapping. Global gene expression patterns are displayed in Figure 5A. As might be expected, with the standard diets, the male and female groups show dissimilar patterns. Interestingly, however, the male group with standard diet defers from the male group with grape-supplemented diet, and the same applies for the respective female groups. Moreover, in terms of hierarchical mapping, the SDGF group shifts away from the SDF group, and aligns more closely with the male group provided with grape-supplemented diet.

As a derivative, Figure 5A,B refine the analysis by organizing genes into ten clusters using k-means clustering. As quantitatively shown by the scale on the right side of the figure, the relative level of enrichment for each of the respective clusters is indicated. As mentioned above, it is of particular interest that the cluster visualization reveals changes that resulted from the dietary administration of grapes in both sexes.

To enhance this analysis further, rather than visualizing global gene expression, heat maps were generated based on differentially expressed genes. The first differential gene expression analysis was performed by comparing SDM vs. SDF and SDGM vs. SDGF. The results are shown in Figure 5C, and then further segregated based on k-means clustering (Figure 5D).

As might be expected from such a comparative analysis, hierarchical mapping most closely links the groups based on sex—SDF with SDGF and SDM with SDGM. However, the question of greatest interest that can be assessed with this dataset is the nature of the changes induced by dietary modification. As shown in Figure 5D, 40 clusters are quantitatively depicted based on color. Employing the original color palette used for creating the scale (comprising 11 colors), and assigning each color a numerical value based on the Euclidean distance between the colors (ranging from −1.5 to +1.5 in intervals of 0.3), each cluster is assigned a quantitative value. Accordingly, the net change for each group (i.e., each column) is calculated, and comparisons between the groups are determined from these results.

Quantitative comparison of the clusters shown in Figure 5D yields the following results: SDF vs. SDM produces a net difference of 7.4 units, whereas SDGF vs. SDGM produces a net difference of 1.4 units. Clearly, there is a much larger difference between the sexes when given the standard diet, and provision of the grape-supplemented diet leads to a convergence. The relative movement by each sex, in an upward and downward manner, leading to this convergence, is not clear.

To examine these changes in more detail, differential gene expression analysis was performed by comparing the SDM vs. SDGM groups and the SDF vs. SDGF groups. As shown in Figure 5E, a notable change is that hierarchical mapping most closely links the groups based on diet (SDGM and SDGF), not sex. Further, using the method described above, the 40 clusters illustrated in Figure 5F were quantitatively analyzed. The results were as follows: SDM vs. SDGM produced a net difference of 4.7 units, and SDF vs. SDGF produced a net difference of 2.7 units. Perhaps by coincidence, the sum of these differences yields a value of 7.4 units, the same as the difference noted above when comparing the SDF vs. SDM groups. In any case, it appears that these alterations led to the most similar case of SDGF vs. SDGM with a difference of only 1.4 units. The ostensible shift in the male phenotype based on diet was greater than the shift in the female phenotype, but in any case, the net movement of gene expression by both sexes provided with a grape-supplemented diet produced a phenotype with greater commonality between the sexes.

### 3.6. Principal Component Analysis

**Figure 6:**
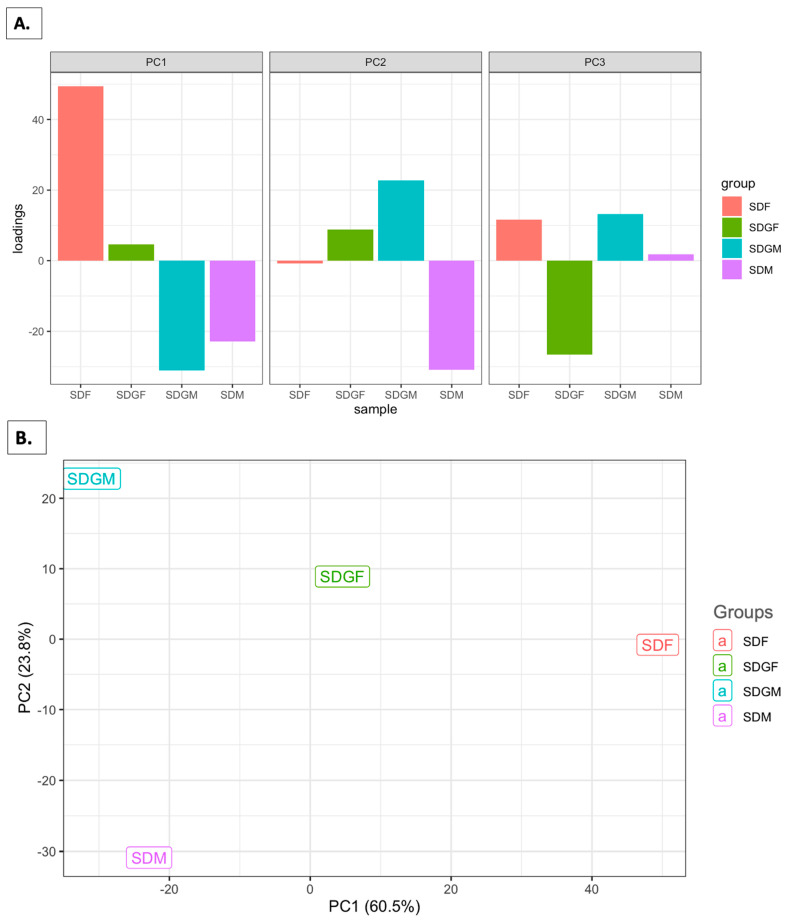

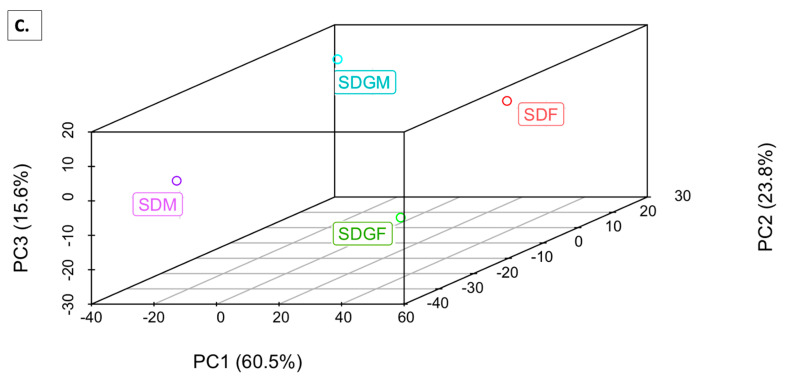
PCA plots for muscles influenced by sex and diet for SDM, SDF, SDGF, and SDGM. (**A**) The bar plot illustrates the loadings of individual variables on the first three principal components (PC1, PC2, and PC3). The bars represent the magnitude and direction of the contribution of the variables by each principal component (PC1, PC2, and PC3). Positive and negative bar heights reflect the positive or negative correlation of the variable with the respective principal components. (**B**) The scatter plot depicts the separation of samples based on their projection onto the first two principal components (PC1 and PC2). The points represent samples, and the position reflects similarities or differences. (**C**) The 3D scatter plot extends the PCA visualization to include PC3 along with PC1 and PC2. Samples are represented as points in a 3D space, with their positions determined by their scores on PC1, PC2, and PC3. Axes labels indicate the percentage of variance contributed by each principal component.

Based on the results shown above, it is obvious that the phenotypic expression of male and female mouse muscles diverges, either with or without the addition of grapes to the diet. However, gross comparison on a macro-phenotypic level does suggest some convergence of male and female muscle when grapes are added to the diet. This idea was accentuated following principal component analysis (PCA). As shown in PCA, small multiple plots in Figure 6A, PC1 and PC2 of the SDGF, clearly resemble those of the SDGM to a much greater extent than those of the SDF. This does not hold for PC3, but the migration of the SDGF toward the male relative to the SDF is clearly illustrated in Figure 6B, where PC1 and PC2 comprise 84.15% of the variation. This is further illustrated in the three-dimensional portrayal shown in Figure 6C, where we see SDGF aligning with SDGM, aside from the influence of the PC3 coordinate.

In the next phase of this comparative analysis, we evaluated the four groups employing KEGG, GO, and Reactome programs.

### 3.7. KEGG Pathways

**Figure 7:**
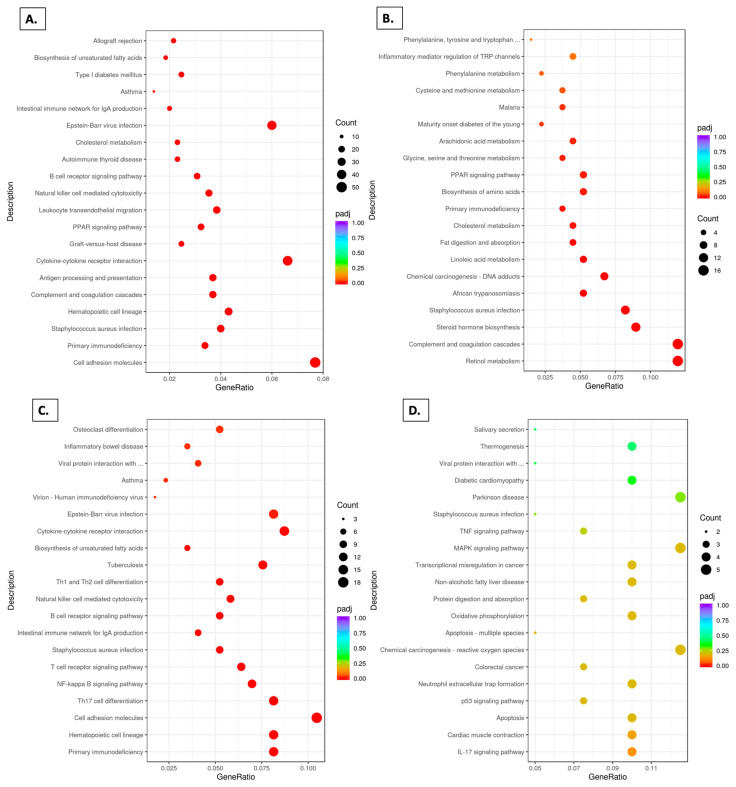

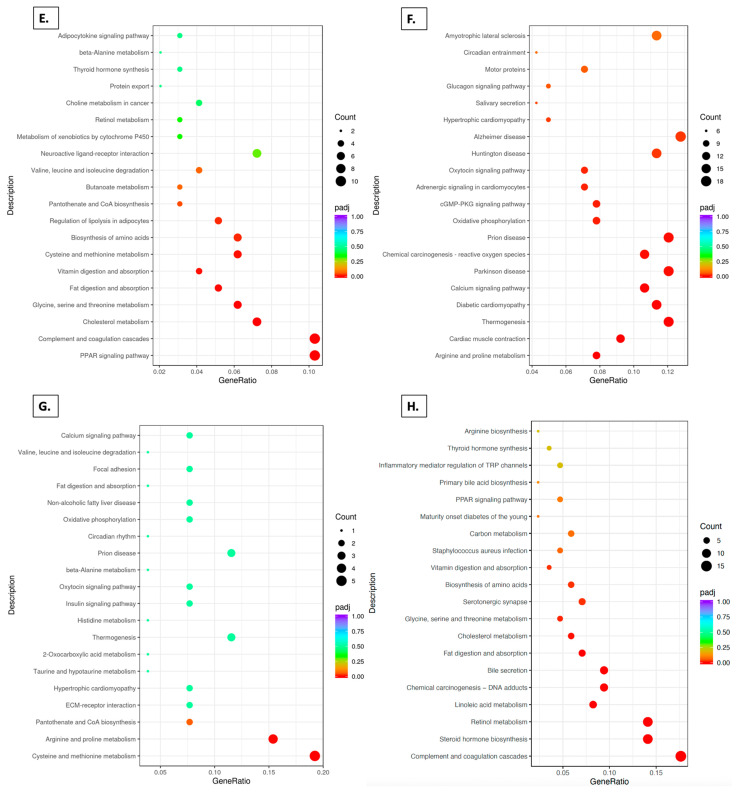
KEGG analysis for comparisons based on standard and grape-supplemented diets. (**A**) SDM vs. SDF down-regulated. (**B**) SDGM vs. SDGF down-regulated, 18 out of the 20 pathways have Padj < 0.05. (**C**) SDGF vs. SDF down-regulated, Padj < 0.05. (**D**) SDGM vs. SDM down-regulated, no significant difference. (**E**) SDGM vs. SDM up-regulated, 10 out of the 20 pathways have Padj < 0.05. (**F**) SDM vs. SDF up-regulated, 17 out of the 20 pathways have Padj < 0.05. (**G**) SDGM vs. SDGF up-regulated, 2 out of the 20 pathways have Padj < 0.05. (**H**) SDGF vs. SDF up-regulated, 12 out of the 20 pathways have Padj < 0.05.

Comparison of SDM vs. SDF using KEGG pathway analysis listed the top 20 pathways in which SDF was enriched relative to SDM (Padj < 0.05) (Figure 7A). In animals receiving the grape diet, there were also 20 top pathways listed in which SDGF was enriched relative to SDGM (Figure 7B), five of which were the same as with the non-grape-fed groups (PPAR signaling, primary immunodeficiency, cholesterol metabolism, *S. aureus* infection, and complement and coagulation cascades). Notably, however, of the remaining 15 pathways showing enrichment of SDGF, relative to SDGM, 7 are likely due to SDF being down-regulated relative to SDGF (Figure 7C), whereas no pathways were significantly down-regulated when comparing SDGM and SDM (Figure 7D). In addition, five of the pathways showing enrichment of SDGF relative to SDGM (Figure 7B), were actually enriched in SDGM relative to SDM (Figure 7E). These data suggest a movement toward convergence in regard to the male and female groups, when grape is added to the diet.

Additional comparisons of the SDM and SDF groups using KEGG analysis identified 20 pathways in which the SDM group was enriched relative to the SDF group (Figure 7F). These differences were largely negated when comparing the SDGM with SDGF groups, wherein only three pathways (Padj < 0.05) were found enriched in the SDGM group relative to the SDGF group (Figure 7G). Of the pathways found enriched in the SDM group relative to the SDF group, but not enriched in the SDGM group relative to the SDGF group, it is likely that five were normalized due to enrichment of the SDGF group versus the SDF group (Figure 7H). Furthermore, of the pathways enriched in the SDM group vs. the SDF group (Figure 7F), but not in the SDGM group vs. the SDGF group (Figure 7G), seven appear on the list of pathways down-regulated in the SDGM group relative to the SDM group (Figure 7D). Although relative changes in these seven pathways did not achieve statistical significance, it is likely that the modest shifts contributed to major shifts observed in the final comparison of the SDGM and SDGF groups (Figure 7G). The only pathway that persisted in terms of being elevated in the SDM and SDGM groups versus the SDF and SDGF groups was arginine and proline metabolism.

In sum, in the situation wherein the SDGF group pathways were enriched relative to the SDGM group pathways (Figure 7B), it appears there was a movement toward normalization in all but nine of the cases, and in the situation wherein the SDGM group pathways were enriched relative to the SDGF group pathways (Figure 7G), all differences were negated, aside from three cases.

### 3.8. GO Domains

**Figure 8:**
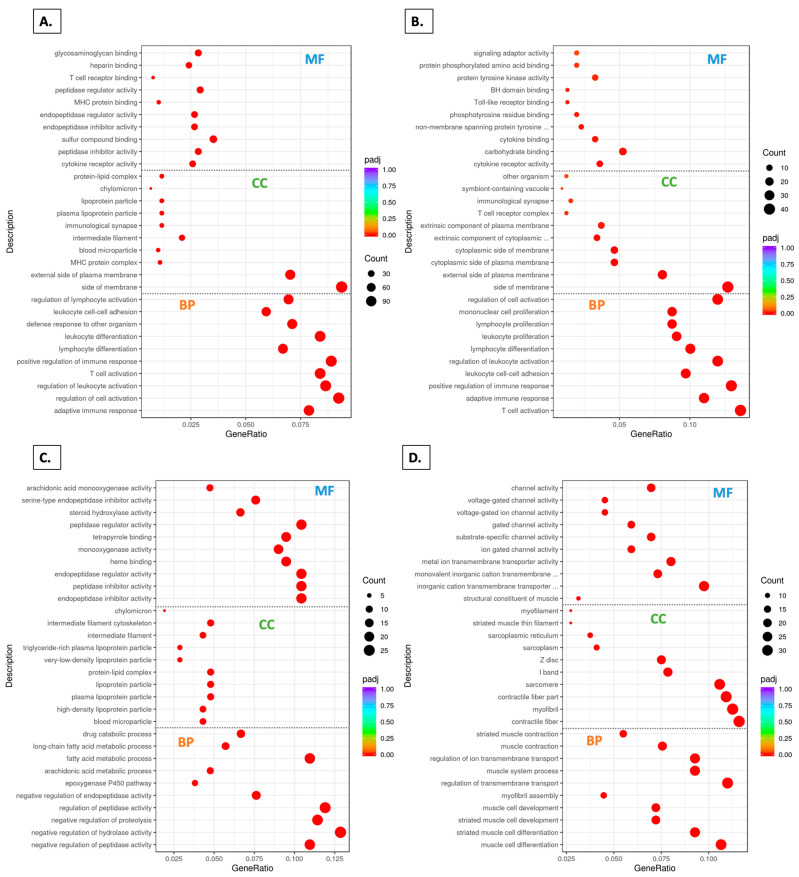

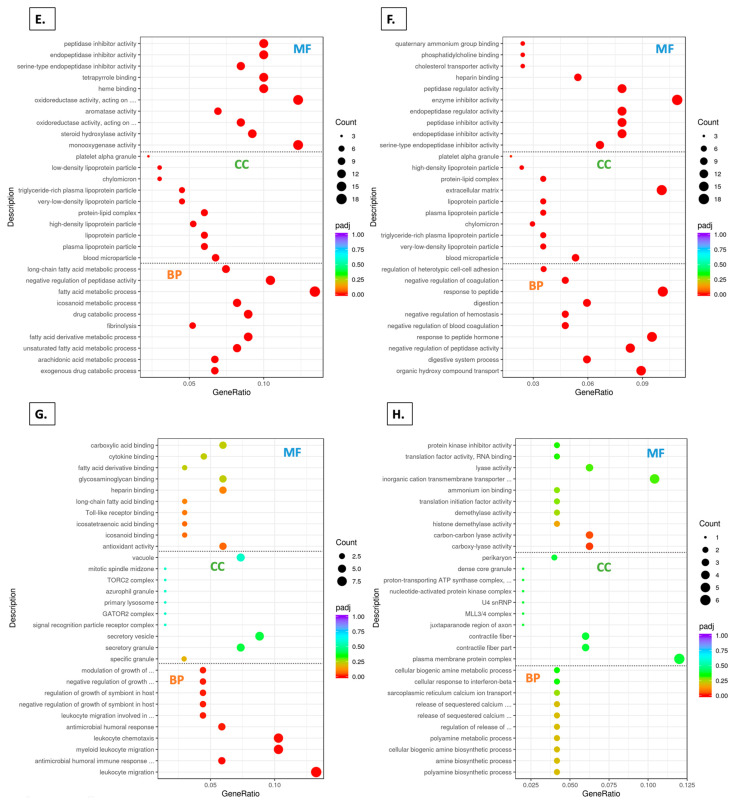
GO term analysis for comparisons based on control and grape-supplemented diets. The plots are divided into three parts represented by the pathways in BP, CC, and MF domains. (**A**) SDM vs. SDF down-regulated, Padj < 0.05. (**B**) SDGF vs. SDF down-regulated, Padj < 0.05. (**C**) SDGM vs. SDGF down-regulated, Padj < 0.05. (**D**) SDM vs. SDF up-regulated, Padj < 0.05. (**E**) SDGF vs. SDF up-regulated, Padj < 0.05. (**F**) SDGM vs. SDM up-regulated, Padj < 0.05. (**G**) SDGM vs. SDM down-regulated; pathways in BP were Padj < 0.05. (**H**) SDGM vs. SDGF up-regulated, where two pathways in MF had Padj < 0.05.

The gene ontology (GO) domains [cellular component (CC), biological process (BP), and molecular function (MF)] revealed a number of changes resulting from grape consumption. In each of the three domains, ten features enriched in the SDF group relative to the SDM group are shown in Figure 8A. In the BP domain, five of the ten features were reduced in the SDGF group relative to the SDF group (Figure 8B). In the CC and MF domains, the SDGF group versus the SDF groups comparisons showed a reduction in three features and one feature, respectively (Figure 8B). Four of the features in the MF domain remained elevated in the SDGF group relative to the SDGM group, five of the features in the CC domain remained elevated, and no features in the BP domain were common (Figure 8C).

Of greatest interest are the data shown in Figure 8D. As might be expected, features specifically related to muscle structure and function listed in all three domains were elevated in the SDM group relative to the SDF group. A number of features were found to significantly differ when comparing the SDGF vs. SDF groups (up-regulated) (Figure 8E), or the SDGM vs. SDM groups (up- or down-regulated) (Figure 8F,G). However, none of the features listed in Figure 8D, comparing the SDM and SDF groups, appeared in these lists (Figure 8E,F or Figure 8G).

In the final comparison, the SDGM and SDGF groups (Figure 8H) showed no significant differences aside from carbon–carbon lyase activity and carbon lyase activity in the MF domain, suggesting that key muscle-related features listed in Figure 8D no longer apply, and the males and females bear great similarity.

### 3.9. Reactome Pathways

**Figure 9:**
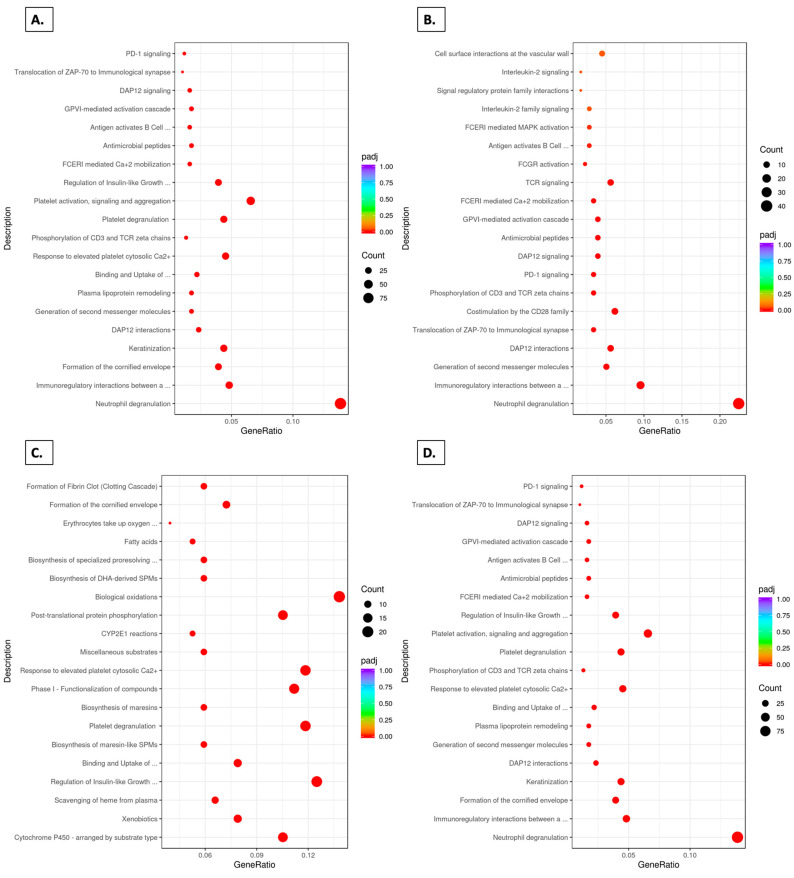

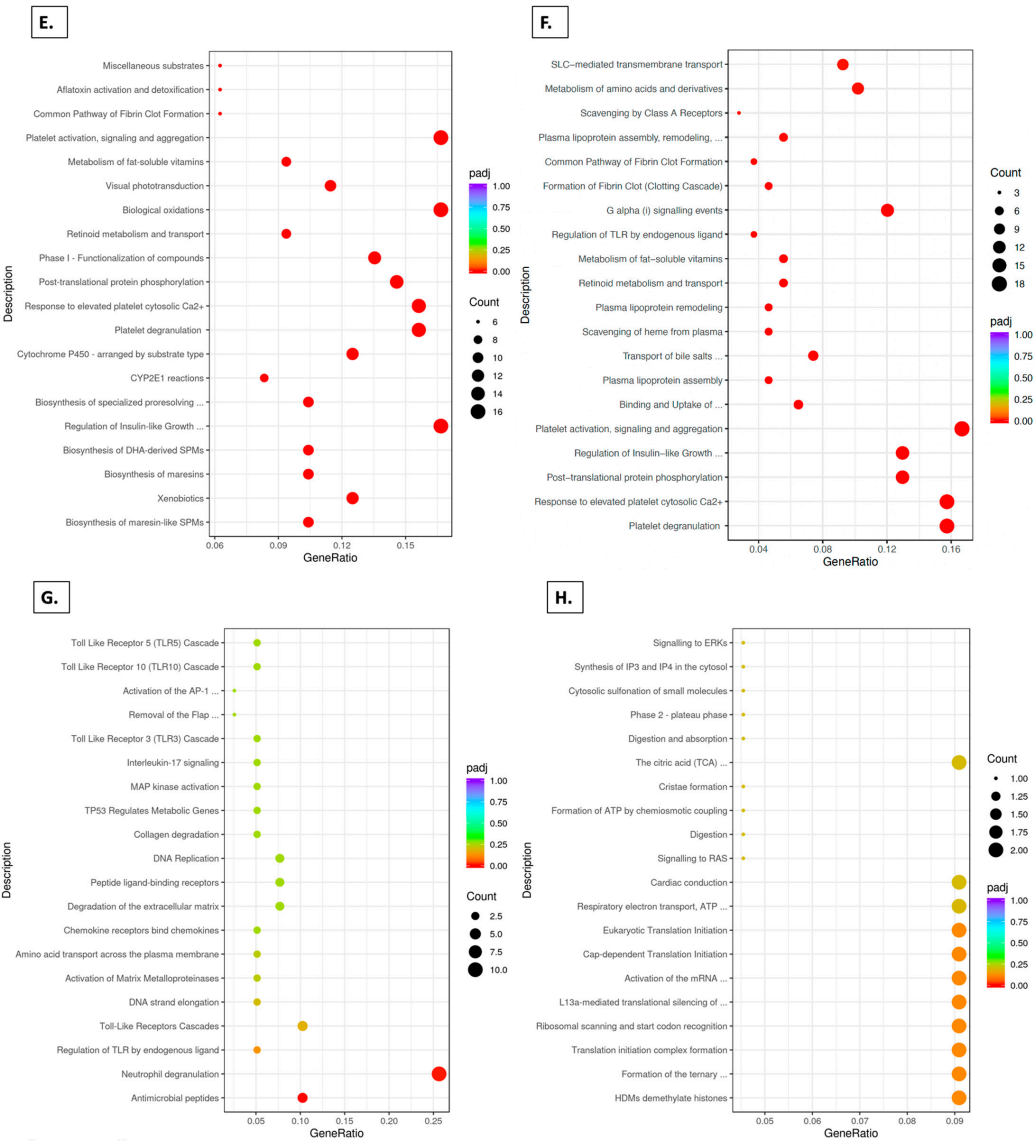
Top 20 Reactome pathway analyses for comparisons based on control and grape-supplemented diets. (**A**) SDM vs. SDF down-regulated, Padj < 0.05. (**B**) SDGF vs. SDF down-regulated, Padj < 0.05. (**C**) SDGM vs. SDGF down-regulated, Padj < 0.05. (**D**) SDM vs. SDF down-regulated, Padj < 0.05. (**E**) SDGF vs. SDF up-regulated, Padj < 0.05. (**F**) SDGM vs. SDM up-regulated, Padj < 0.05. (**G**) SDGM vs. SDM down-regulated, where 2 out of the 20 pathways were under Padj < 0.05. (**H**) SDGM vs. SDGF up-regulated, no significant difference.

As shown in Figure 9A, 20 top Reactome pathways were enriched in the SDF group relative to the SDM group. Of these 20 pathways, 12 were reduced in the SDGF group relative to SDF group (Figure 9B), and 4 of the features remained elevated when comparing the SDGF with SDGM groups (Figure 9C).

Of greatest interest are the data shown in Figure 9D. When comparing the SDM and SDF groups, six features were enriched in the SDF group, including muscle contraction and striated muscle contraction. A number of features were up-regulated in the SDGF group relative to the SDF group (Figure 9E), and the SDGM group relative to the SDM group (Figure 9F), but none of these features appeared in the comparison of the SDM group and the SDF group (Figure 9D). Only two features were found to be enriched in the SDM group relative to the SDGM group (Figure 9G), but again, these were not common with those observed when comparing the SDM and SDF groups (Figure 9D).

The final comparison the SDGM group with the SDGF group (Figure 9H), showed modest differences in some Reactome pathways, but no significant differences. These results suggest that the key muscle-related differences in which the SDF group was enriched relative to the SDM group (Figure 9D) no longer exist when the grape diet was administered, and the males and females bear great similarity.

### 3.10. Genetic Expression in Different Groups

To examine gene expression changes from both sex-based comparisons (Figure 5D) and grape diet vs. non-grape diet comparisons (Figure 5F), we analyzed specific clusters identified in heatmaps generated from the following pairwise comparisons: SDM vs. SDF and SDGM vs. SDGF (Figure 5D), as well as SDGM vs. SDM and SDGF vs. SDF (Figure 5F). By consolidating these genes into a single panel, we could probe their expression from both perspectives.

**Figure 10:**
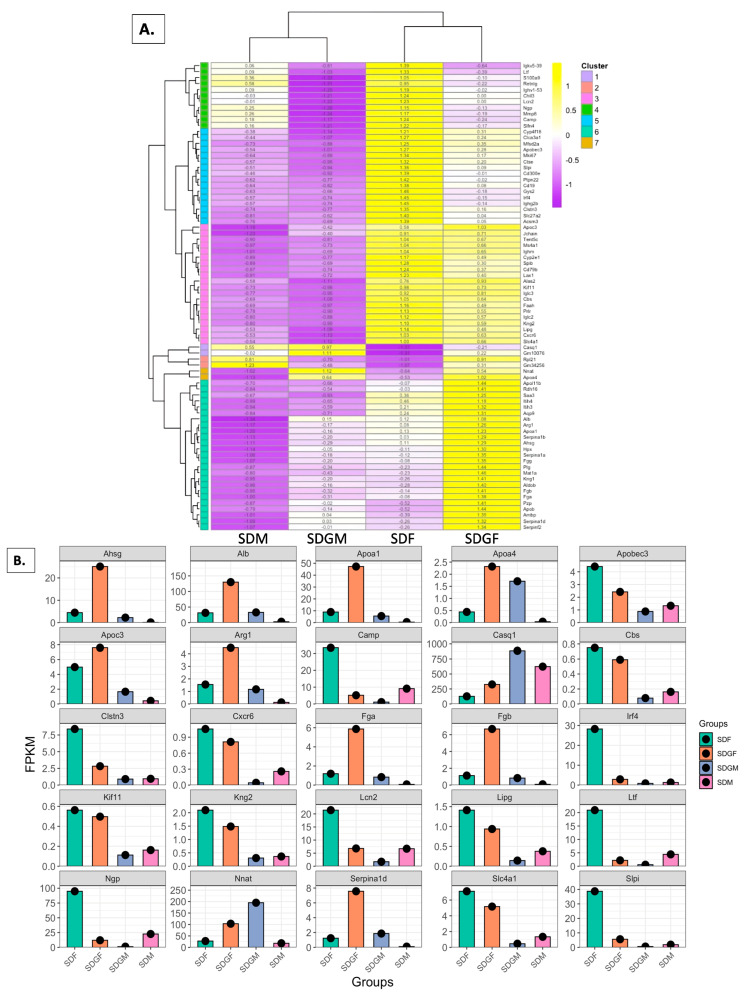
(**A**) Heatmap depicting gene expression from the DEG list of SDM, SDGM, SDF, and SDGF groups. Genes were selected based on distinct clusters identified in Figure 5D (clusters 2, 3, 4, 5, 6, 7, 9, and 10) and Figure 5F (clusters 2, 5, 6, 7, 8, and 9). Z-scores of these genes were calculated and compared across the four groups. For hierarchical clustering, the dendrogram was divided into seven clusters. (**B**) Heatmap-derived selection of 25 genes including *Ahsg*, *Alb*, *Apoa1*, *Apoa4*, *Apobec3*, *Apoc3*, *Arg1*, *Camp*, *Casq1*, *Cbs*, *Clstn3*, *Cxcr6*, *Fga*, *Fgb*, *Irf4*, *Kif11*, *Kng2*, *Lcn2*, *Lipg*, *Ltf*, *Ngp*, *Nnat*, *Serpina1d*, *Slc4a1*, and *Slpi* identified as differentially expressed and relevant to muscle function. The expression levels are reported in FPKM values across the four groups.

From Figure 5D, clusters 2, 3, 4, 5, 6, 7, 9, and 10 were selected, while from Figure 5F, clusters 2, 5, 6, 7, 8, and 9 were visually distinct. The *z*-scores of these genes were then compared across the SDM, SDGM, SDF, and SDGF groups, resulting in the data shown in Figure 10A.

To further investigate the roles of these genes, we analyzed their individual functions, in general, considering relationships to muscle. This led to the recognition of 25 genes with muscle-specific functions that showed visual differences in the heatmap (Figure 10A). The selected genes were *Ahsg*, *Alb*, *Apoa1*, *Apoa4*, *Apobec3*, *Apoc3*, *Arg1*, *Camp*, *Casq1*, *Cbs*, *Clstn3*, *Cxcr6*, *Fga*, *Fgb*, *Irf4*, *Kif11*, *Kng2*, *Lcn2*, *Lipg*, *Ltf*, *Ngp*, *Nnat*, *Serpina1d*, *Slc4a1*, and *Slpi*. Expression levels of each of these genes, expressed as FPKM values, are shown in Figure 10B for each of the four groups of mice.

First of all, it is notable that in 24 of the 25 cases, the FPKM values of the SDF group were higher than the corresponding values in the SDM group. A comparison of the SDF group with the SDGF group indicated up- and down-regulation of 11 and 14 genes, respectively. In the male groups, changes were generally more modest, but the grape diet resulted in 11 genes being up-regulated, 10 genes being down-regulated, and 4 genes showing little change relative to the control diet. In most cases, the upward or downward movement of a specific gene was the same with males and females (up-regulation, 11; down-regulation, 11). In four cases wherein the movement of a specific gene in the female was downward, the level in the male was low in both the SDM and SDGM groups, with no appreciable change.

Prominent differences between the SDF and SDM groups were negated in some cases when comparing the SDGF and SDGM groups (*Camp*, *Clstn3*, *Irf4*, *Ltf*, *Ngp*, *Slpi*) and accentuated in others (*Ahsg*, *Alb*, *Apoa1*, *Arg1*, *Fga*, *Fgb*, *Serpina1d*). Comparable levels were observed between the SDF and SDM groups in two cases (*Alb*, *Nnat*), but this was increased to ten cases when comparing the SDGF and SDGM groups (*Apoa4*, *Apobec3*, *Camp*, *Clstn3*, *Irf4*, *Lcn2*, *Ltf*, *Ngp*, *Nnat*, *Slpi*).

In females, comparing the SDF vs. SDGF groups, there were sharp increases in *Ahsg* (alpha-2-Heremans–Schmid glycoprotein), *Alb* (albumin), *Apoa1* and *4* (apolipoprotein 1 and 4), *Apoc3* (apolipoprotein C-III), *Arg1* (arginase 1), *Casq1* (calsequestrin 1), *Fga* (codes for fibrinogen alpha chain), *Fgb* (codes for fibrinogen beta chain), *Nnat* (neuronatin), and *Serpina1d* (production of serine protease inhibitor, serpin) as a result of grape consumption. Aside from *Apoa4*, there were corresponding increases in all of these FPKM values in males as a result of grape consumption, although the changes were not as profound.

In regard to decreases in FPKM values, when comparing the SDF vs. SDGF groups, there were sharp reductions in *Apobec3* (apoliprotein B mRNA editing enzyme, catalytic peptide 3), *Camp* (cathelicidin antimicrobial peptide), Cltin3 (calsyntenin-3), *Irf4* (interferon regulatory factor 4), *Kng2* (kininogen 2), *Lcn2* (lipicalin 2), *Lipg* (endothelial lipase), *Ltf* (lactotransferrin), *Ngp* (neutrophilic granule protein), and *Slpi* (secretory leucocyte protease inhibitor) as a result of grape consumption. As above, with decreasing FPKM values, there were corresponding decreases (or no change) in all of the respective values in males as a result of grape consumption, although the changes were more modest.

Some functional aspects of these genes are listed in Table 2 and described in greater detail below (Discussion). Briefly, *Ahsg* is associated with insulin sensitivity and the lean muscle mass regulation. *Alb* functions as a reservoir for dietary amino acids and is linked to muscle function, with lower levels associated with sarcopenia. *Apoa1* and *Apoa4* enhance glucose uptake and reduce fat accumulation, with *Apoa1* activating the IR/IRS-1/PI3K/Akt/AS160 pathway in skeletal muscle, and *Apoa4* enhancing glucose uptake in cardiac and adipose tissues. *Apoc3* is implicated in ER stress and inflammation, though its role in muscle is secondary to its function in lipid metabolism. *Arg1* regulates oxidative stress in degenerative diseases, while *casq1* is critical for calcium homeostasis in muscle contraction.

Conversely, genes downregulated by grape consumption suggest physiological adaptations in muscle. *Apobec3* is typically absent in muscle but may indicate pathology when highly expressed. *Camp* is associated with muscle degeneration, while *clstn3* is involved in thermogenesis and glucose metabolism. *Irf4* influences exercise capacity, obesity, and insulin resistance, with increased expression observed in obese individuals. *Kng2* and *lcn2* are linked to inflammatory responses and muscle integrity, while *ltf* may aid in muscle repair. *Ngp* modulates inflammatory pathways, and *slpi* is involved in tissue homeostasis, with an increased expression suggesting metabolic stress.

There were no cases in which the FPKM values were increased in one sex and decreased in the other sex as a result of grape consumption, or vice versa. There was only one example in which the FPKM value was higher in the male than the female, *casq1* (calsequestrin 1), in both the standard diet and grape-supplemented diet groups. In skeletal muscle, *casq1* plays a role in calcium buffering to maintain the sarcoplasmic reticulum with a suitable amount of calcium [41].

## 4. Discussion

Consumption of fruits and vegetables has long been known to modulate the expression of specific genes capable of reducing the risk of disease. Some examples include proinflammatory genes (such as ICAM1, IL1R1, IL6, TNF-α, and NFĸB), p53, PTEN, and detoxification genes (including phase I cytochrome P450 enzymes, phase II conjugation enzymes, Nrf2 signaling, and metallothionein) [42]. Some interactions with medications are also well known, as exemplified by grapefruit [43]. More recently, the broader impact of diet on mammalian homeostasis has been recognized [30,44,45].

In 2022, global consumption of table grapes was estimated to be 32.6 metric tons. Total production of all fresh grapes, including wine and dried, was estimated to be 80.1 metric tons [46]. Production and consumption vary year by year, but, clearly, human ingestion of this magnitude warrants investigation into its impact on health. We have been particularly interested in the potential of dietary table grapes to mediate ‘omic’ responses in mice and humans, and analyses of corresponding implications. Using mice as a model system, gene expression is modulated in multiple tissues as a result of grape consumption [18], as well as metabolomic alterations [17,22]. In humans, grape consumption leads to alterations in the microbiome [17]. In the current work, our objective was to determine potential changes in gene expression using elderly mice as a model. The progressive loss of muscle function in humans, particularly at the age of 60 years or more, has detrimental effects on the quality of life [1]. The age of the mice employed in this study can be roughly equated to a human age of 80 years [28].

As previously reported [19], there were no discernable differences in the respective group weights of the male or female mice irrespective of dietary grape supplementation, nor were there any significant differences in the weight of hip muscle. Here, we report histopathological evaluation of muscle tissue from all designated groups, using both H&E and Masson’s trichrome stains. Both male and female mice on the standard diet exhibited predominantly normal muscle histology, with only minimal chronic perivascular inflammation in one male mouse sample, which is not considered a significant pathological finding. Similarly, male and female mice receiving the grape-supplemented diet also displayed normal muscle histology, with no evidence of significant pathological changes. Overall, no profound histopathological differences were seen between the groups.

So, in terms of gross morphology, the groups were not differentiated, neither based on sex nor diet. However, we thought it would be of interest to investigate some intrinsic characteristics of the muscle tissues to further define the gross similarities, or, potentially, differences based on sex, and differences resulting from the dietary consumption of grapes. The primary method employed was RNA-Seq. Our supposition is that changes at the level of gene expression will lead to downstream changes in physiology and function.

By examining over 20,000 gene transcripts, as illustrated by Venn diagrams, volcano plots, and heatmaps, it is abundantly clear that the SDF group can be differentiated from the SDGF group, the SDM group can be differentiated from the SDGM group, and the respective female groups can be differentiated from the respective male groups. There is certainly no congruent phenotypic expression pattern between any of the groups. Bearing this in mind, however, the focal point of this work was to assess the changes induced by grape supplementation of an otherwise ‘bland’ diet and attempt to decipher any functional significance.

Unexpectedly, beginning with the differential examination of gene clusters produced by the four groups of mice, it started to become clear that the net movement of gene expression by both sexes provided with a grape-supplemented diet produced a phenotype with greater commonality between the sexes. This idea was further accentuated by principal component analysis (PCA). It was found that PC1 and PC2 of the SDGF group evidently resembled those of the SDGM group to a much greater extent than they did those of the SDF group.

In further support of this notion, when examining the top 20 KEGG pathways, comparison of the SDF group with the SDM group revealed all 20 were significantly different, whereas comparison of the SDGF group with the SDGM group revealed only 3 of the top 20 were significantly different. In a similar vein, analysis of the top 10 GO terms in the domains of MF, CC, and BP demonstrated all ten features in each domain were significantly different when comparing the SDF and SDM groups. However, when comparing the SDGF and SDGM groups, only three features in the MF domain were significantly different, and no features in the CC and BP domains were significantly different. Finally, congruent results were obtained through Reactome pathway analysis. Again, examining the top 20 pathways, all 20 were significantly different when comparing the SDF and SDM groups, and there were no significant differences in the top 20 Reactome pathways when comparing the SDGF and SDGM groups.

As an attempt to inspect this congruity in more granular detail, we selected 25 differentially expressed genes for further analysis. Expression levels of each respective group were expressed as FPKM values. Of the 25 selected DEGs, 11 were sharply up-regulated as a result of grape consumption. A brief synopsis of the reported functions of these genes follows.

*Ahsg* is suggested to be important for the regulation of body fat and insulin sensitivity [47]. Perhaps more importantly, it is associated with lean muscle mass [48].

*Alb* serves as a reservoir of excessive dietary amino acids that is protected from irreversible oxidation [49]. In muscle, lower levels are associated with decline in function [50] and sarcopenia [51].

*Apoa1* and *4* improve glucose uptake and reduce fat accumulation [52]. Apoa1 increases glucose disposal in skeletal muscle by activating the IR/IRS-1/PI3K/Akt/AS160 signal transduction pathway [53]. Apoa4 lowered fasting blood glucose in both WT and diabetic KKAy mice by increasing glucose uptake in cardiac muscle, white adipose tissue, and brown adipose tissue through a mechanism that was partially insulin-independent [54].

*Apoc3* in skeletal muscle from transgenic mice overexpressing apoCIII showed increased levels of some ER stress and inflammatory markers and increased phosphorylated ERK1/2 levels, whereas PGC-1α levels were reduced [55]. *Apoc3* can inhibit lipoprotein lipase thereby leading to increased triglyceride levels [56], but its role in muscle is not considered significant relative to its function in the liver, for example.

*Arg1.* Arginine is a semi-essential amino acid which is the substrate for both nitric oxide synthase (NOS) and arginase enzyme. Arg1 is known to regulate oxidative stress in various degenerative diseases by modulating nitric oxides (NO) [57,58,59].

*Casq1* plays a key role in maintaining calcium levels for muscle contraction [41]. It serves as a major Ca^2+^-buffering protein to maintain the sarcoplasmic reticulum (SR) with a suitable amount of Ca^2+^ at each moment, a dynamic Ca^2+^ sensor in the SR that regulates Ca^2+^ release from the SR to the cytosol, a structural regulator for the proper formation of terminal cisternae, and a reverse-directional regulator of extracellular Ca^2+^. Of note, the diminution of casq1 resulting from mutation is associated with human skeletal muscle diseases [60].

*Fga* and *Fgb*. Coding for fibrinogen alpha and beta chains, a relationship to muscle is not apparent. However, a preliminary report indicated function as “hub” genes (i.e., genes involved in activating other genes) in muscle of aged mice [61].

*Nnat* is involved in adaptive thermogenesis. It is a transmembrane protein in the endoplasmic reticulum involved in metabolic regulation. It shares sequence homology with sarcolipin (SLN), which negatively regulates the sarco(endo)plasmic reticulum Ca^2+^-ATPase (SERCA) that maintains energy homeostasis in the muscles. Regulation by dietary fat has been reported as well [62].

*Serpina1d* increases with exercise [63], may play a role in muscle contraction [64], and promotes muscle hypertrophy due to inhibiting the negative regulator of muscle growth, myostatin [65].

Conversely, decreases in FPKM values in conjunction with reported functional aspects of the respective genes allow for some suggestions regarding alterations in the muscle physiology that result from this action of grape consumption. Of the 25 selected DEGs, 10 were sharply down-regulated. A brief synopsis of reported functions follows.

*Apobec3*. Apoliprotein B mRNA editing enzyme, catalytic peptide 3 counteracts the replication of retroviruses [66]. It is generally not expressed in muscle, although high levels could be associated with pathology [67].

*Camp* is involved in muscle degeneration and can be implicated in Duchenne muscular dystrophy [68].

*Clstn3.* Calsyntenin 3b (Clstn3b) is a novel gene that promotes brown fat thermogenesis in mice [69]. It is associated with obesity risk and adipose tissue dysfunction [70]. Expression is inversely correlated with certain parameters of whole-body and adipose glucose metabolism, including fasting plasma glucose (FPG), fasting insulin (FINS), glycosylated hemoglobin A1c (HbA1c), adipose tissue insulin resistance (Adipo-IR), and homeostasis model assessment of insulin resistance (HOMA-IR) [71].

*Irf4*. Knockout mice show better exercise capacity and increased glycogen content, whereas the opposite applies with Irf4 overexpression [72]. IRF4 plays an important role in the development of obesity and insulin resistance and regulates skeletal muscle amino acid metabolism [73]. Expression is significantly increased in the muscles of obese subjects as well as in the gastrocnemius muscles from two different animal models of obesity [diet-induced obesity (DIO) and *db*/*db* mice] [74].

*Kng2*. Kininogens, glycoproteins that serve as a high molecular weight precursor of kinins (such as bradykinin), may be released during inflammatory conditions [75].

*Lcn2*. Increased levels are associated with hallmarks of Duchenne muscular dystrophy, and, in mice, reduced levels lead to better grip strength, increased intact muscle, and reduced serum creatine kinase [76]. Furthermore, omics analysis performed on the longissimus dorsi (LD) muscle explanted from mice subjected to spaceflight shows up-regulation of *Lcn2* compared to earth gravity control [77].

*Lipg* is predominantly expressed by vascular endothelial cells, macrophages, and smooth muscle cells and may not have a major function in skeletal muscle [78]. Endothelial lipase (*Lipg*) is a cell-surface-associated lipase that displays phospholipase A1 activity towards phosphatidylcholine present in high-density lipoproteins (HDL) [79].

*Ltf* may play a role in the repair of skeletal muscle damage. As a naturally active glycoprotein, it possesses anti-inflammatory, antimicrobial, antitumor, and immunomodulatory activity [80].

*Ngp* interacts with the complex of LPS and LPS-binding protein (LBP). This interaction blocks the binding of the complex of LPS and LBP to TLR4 and the downstream inflammatory signals [81].

*Slpi* maintains tissue homeostasis by preventing protease-induced tissue damage. However, higher levels may be indicative of an alarm function [82], suggesting induction would be the result of some type of metabolic stress. *Slpi* has been broadly studied in fields such as wound repair, infection, growth, and cell proliferation [83].

Four additional genes were also decreased as a result of grape consumption, though less drastically, including *Cbs* (cystathionine beta-synthase), *Cxcr6* (CXC motif chemokine receptor 6), *Kif11* (kinesin family member 11), and *Slc4a1* (anion exchanger 1). It should be noted, however, that although modest reductions were observed when given the grape diet, relatively high levels were still retained. Of the 25 selected DEGs, a brief synopsis of the reported functions of the four genes showing modest reduction follows.

*Cbs* plays a critical role in regulating homocysteine levels which can be detrimental to muscle health. Mutations in the gene can lead to muscle weakness and other complications [84].

*Cxcr6* is a seven-transmembrane domain G-protein-coupled receptor, predominantly expressed on activated CD4+, CD8+ T cells, NK cells, and NKT cells [85]. CXC chemokine receptor 6 (CXCR6), a seven-transmembrane domain G-protein-coupled receptor, plays a pivotal regulatory role in inflammation and tissue damage through its interaction with CXC chemokine ligand 16 (CXCL16). This axis is implicated in the pathogenesis of various fibrotic diseases and correlates with clinical parameters that indicate disease severity, activity, and prognosis in organ fibrosis [85]. The presence in muscle is due to resident T-cell signature in cases of idiopathic inflammatory myopathies (IMM) [86].

*Kif11* plays a role in regulating cell survival and proliferation. Inhibiting the expression and activity of Kif11 can lead to cell cycle arrest, reduced proliferative capacity, and the initiation of apoptosis [87,88]. In vascular injury, it has been suggested that inhibition may be a strategy to counteract neointimal formation [89]. It has also been suggested that *Kif11* is implicated in promoting protein secretion and cellular transport [90].

*Slc4a1* is anion exchanger protein expressed in kidney and red blood cells. Deficiency (e.g., mutation) is associated with distal renal tubular acidosis [91]. It has been suggested that oxidative damage of red blood cell *Slc41* is associated with detrimental consequences in oxidative-stress-related diseases including inflammation, metabolic dysfunctions, and aging [92].

These alterations in FPKM values, either upward or downward, in conjunction with reported functional aspects of the respective genes, allow for some suggestions regarding alterations in the muscle physiology that result from grape consumption. In brief, elevated levels are associated with lean muscle mass (*Ahsg*), prevention of oxidation muscle decline and sarcopenia (*Alb*), improved glucose uptake and reduced fat accumulation (*Apoa1* and *4*), regulation of oxidative stress (*Arg1*), maintenance of calcium levels for muscle contraction (*Casq1*), adaptive thermogenesis (*Nnat*), and muscle contraction (*Serpina1d*).

Conversely, some implications regarding decreases in FPKM values when provided with the grape diet are as follows. In cases where high levels are associated with a function, it is presumed that lower levels, as would result from down-regulation, imply the opposite. In this context, high levels of *Camp* and *Lcn2* are associated with muscle degeneration and can be implicated in Duchenne muscular dystrophy; *Clstn3* is associated with obesity risk; and *Irf4* knockout mice show better exercise capacity. Down-regulated levels of some other genes suggest reduced necessity for counteracting viral replication (*Apobec3*), reduced inflammation (*Kng2, Ltf*, and *Ngp*), and metabolic stress (*Slpi*).

While it is clear that changes in gene expression are extensive as the result of grape consumption, the mechanisms yielding these alterations remain to be defined. Based on the experimental design, it is reasonable to conclude the responses are due to the phytochemical components of the grape. These components may regulate gene expression through changes in the chromatin structure (including DNA methylation and histone modification), non-coding RNA, activation of transcription factors by signaling cascades, or direct ligand binding to the nuclear receptors [42]. Additional elements that may come into play include the microbiome and metabolome [93]. Indeed, the situation is likely very complex. A deeper understanding of these possibilities may help to explain the sex differences currently reported.

Finally, although we consider the mouse model currently employed as physiologically relevant, human trials are ultimately required for definitive proof. In one study, supplemental grape consumption was reported to have no discernable effect when administered to recreationally active young adults [94]. For example, there were no significant effects on maximal oxygen consumption (VO2max), work capacity, mood, perceived health status, inflammation, or pain. In our view, this investigation has little bearing on the current work since it is not surprising that recreationally active young adults would even require any type of remedial care.

Of greater interest will be the results of an ongoing study in which postmenopausal women will be provided with the same grape product used in the current work and evaluated for improvement in muscle function [95]. Further, it is known that grape administration modulates gene expression in humans [96], as well as the human microbiome [17]. In sum, we have good confidence in our model system and its applicability to the human situation.

## 5. Conclusions

Loss of skeletal muscle mass and function, sarcopenia, is a well-known consequence of aging [1,97]. Here, we investigated the potential of long-term dietary ingestion of grapes to influence gene expression in the skeletal muscle of male and female mice evaluated when they reached an age that corresponds with approximately 80 years in a human. Key findings are summarized in Table 3. Relative to a ‘bland’ diet, we discerned a much stronger congruence of gene expression in females and males provided with the grape diet. Moreover, this congruence was linked to equivalence in metabolic pathways (KEGG, Reactome), biological processes, cellular locations, and molecular functions (GO). The upward and downward movements of DEGs were indicative of beneficial effects, applicable to both sexes. In sum, the results strongly suggest the phytochemical constituents of grapes are beneficial for muscle health, especially in females. Naturally, additional work is required to determine if such a homogenic response applies to human beings.

## Figures and Tables

**Table 1:** Constituents of the diets: 4% fat standard diet (SDM and SDF, TD.160157); standard diet with grape powder (SDGM and SDGF, TD.160158); diets were produced by Envigo (Madison, WI, USA).

	Standard Diet (SD) (TD.160157) ^3^	Standard Diet with Grape Powder (SDG) (TD.160158) ^3^
**Formula (g/kg)**		
Casein	195	192.9
DL-Methionine	3.0	3.0
Sucrose	191.1	191.0
Dextrose, anhydrous	66.45	44.3
Fructose	66.45	44.3
Corn starch	235.03	232.88
Maltodextrin	100	100.0
Anhydrous milkfat ^1^	30	29.85
Soybean oil	10	10
Cellulose	50	50
Mineral mix, AIN-76 (170915)	35	35
Potassium citrate, monohydrate	4.03	2.69
Calcium carbonate	4.0	4.0
Vitamin mix, Teklad (40060)	10.0	10.0
Ethoxyquin, antioxidant	0.04	0.04
Grape powder, freeze-dried ^2^	0	50

^1^ For each 100 g of anhydrous milkfat: total fat, 99.8 g; saturated fat, 67 g; *trans* fat, 2.6 g; polyunsaturated fat, 3.9 g; monounsaturated fat, 26.3 g. ^2^ Grape powder is considered to contain 3.71 kcal/g, 3% fat, 88.6% carbohydrate (as a 1:1 mixture of fructose and glucose), 3.58% protein, and 9.73 g/kg K^+^. ^3^ Formulated to 3.6 kcal/g (protein, 19.1%; carbohydrate, 70.5%; fat, 10.4%).

**Table 2:** Functional roles of differentially expressed genes in skeletal muscle following grape consumption.

Interaction Category	Genes Involved	Key Relationship
Muscle Growth and Repair	*Serpina1d*, *Fga*, *Fgb*, *Nnat*, *Ltf*, *Ngp*	Promote muscle growth, repair damage, and reduce inflammation.
Metabolism and Insulin Sensitivity	*Ahsg*, *Apoa1*, *Apoa4*, *Irf4*, *Clstn3*	Improve glucose uptake, reduce fat, and regulate obesity/insulin resistance.
Inflammation and Stress	*Kng2*, *Ltf*, *Ngp*, *Arg1*, *Cbs*	Regulate inflammation, oxidative stress, and tissue repair.
Calcium and Muscle Function	*Casq1*, *Alb*	Maintain calcium levels for muscle contraction and overall muscle health.

**Table 3:** Summary of key findings.

	Key Findings	Observations
1.	No significant differences observed in the histopathological evaluation.	No significant pathological finding
2.	The SDGF group shows a distinct shift compared to the SDF group.	Observed in hierarchical map in the heatmap
3.	Convergence in the grape-supplemented diet groups across both sexes.	Quantitative comparison of the clusters in the heatmap and the PCA plots
4.	Fewer differences were observed in the pathway analysis between the grape diet groups (SDGM vs. SDGF) compared to the standard diet groups (SDM vs. SDF).	Pathway analysis revealed fewer significant differences in the comparison of the grape diet groups to the standard diet groups
5.	Grape supplementation was associated with elevated gene expression levels.	*Ahsg*, *Alb*, *Apoa1* and *4*, *Arg1*, *Casq1*, *Nnat*, and *Serpina1d*
6.	Decreased FPKM values observed with a grape diet.	*Camp*, *Lcn2*, *Clstn3*, *Irf4*, *Apobec3*, *Kng2*, *Ltf*, *Ngp*, and *Slpi*

## Data Availability

The data presented in this study are available in the National Center for Biotechnology Information (NCBI) repository at Bio-project accession number PRJNA1200816.

## Abbreviations

DEG	differential expressed gene
FPKM	Fragments Per Kilobase of transcript per Million mapped reads
Gene symbols	
*Ahsg*	alpha-2-Heremans–Schmid glycoprotein
*Alb*	albumin
*Apoa1* and *4*	apolipoprotein 1 and 4
*Apoc3*	apolipoprotein C-III
*Apobec3*	apoliprotein B mRNA editing enzyme, catalytic peptide 3
*Arg1*	arginase 1
*Camp*	cathelicidin antimicrobial peptide
*Casq1*	calsequestrin 1
*Cltin3*	calsyntenin-3
*Fga*	codes for fibrinogen alpha chain
*Fgb*	codes for fibrinogen beta chain
*Irf4*	interferon regulatory factor 4
*Kng2*	kininogen 2
*Lcn2*	lipicalin 2
*Lipg*	endothelial lipase
*Ltf*	lactotransferrin
*Ngp*	neutrophilic granule protein
*Nnat*	neuronatin
*Serpina1d*	production of serine protease inhibitor, serpin
*Slpi*	secretory leucocyte protease inhibitor
GO	gene ontology
CC	cellular component
BP	biological process
MF	molecular function
KEGG	Kyoto Encyclopedia of Genes and Genomes
PCA	principal component analysis
SDF	standard diet females
SDGF	standard diet containing 5% grape powder females
SDGM	standard diet containing 5% grape powder males
SDM	standard diet males
